# Enhanced electrical and thermal properties of semi-conductive PANI-CNCs with surface modified CNCs†

**DOI:** 10.1039/d0ra10663a

**Published:** 2021-03-19

**Authors:** Po-Yun Chen, Chieh Hsu, Manikandan Venkatesan, Yen-Lin Tseng, Chia-Jung Cho, Su-Ting Han, Ye Zhou, Wei-Hung Chiang, Chi-Ching Kuo

**Affiliations:** Institute of Organic and Polymeric Materials, Research and Development Center of Smart Textile Technology, National Taipei University of Technology Taipei 10608 Taiwan kuocc@mail.ntut.edu.tw ppaul28865@mail.ntut.edu.tw +886-2-27317174 +886-2-27712171 ext. 2407; Institute of Microscale Optoelectronics, Shenzhen University Shenzhen P. R. China; Institute for Advanced Study, Shenzhen University Shenzhen P. R. China; Department of Chemical Engineering, National Taiwan University of Science and Technology 10607 Taipei Taiwan

## Abstract

Cellulose nanocrystals (CNCs) are the most commonly used natural polymers for biomaterial synthesis. However, their low dispersibility, conductivity, and poor compatibility with the hydrophobic matrix hinder their potential applications. Therefore, we grafted sulfate half-ester and carboxylic functional groups onto CNC surfaces (S-CNC and C-CNC) to overcome these shortcomings. The effect of the dopants, surfactant ratios, and properties of CNCs on the thermal stability, conductivity, and surface morphology of polyaniline (PANI)-doped CNC nanocomposites were investigated through emulsion and *in situ* polymerization. The higher electrical conductivity and well-dispersed morphology of SCNC–PANI_30_ (1.1 × 10^−2^ S cm^−1^) but lower thermal stability than that of CCNC–PANI_30_ (*T*_0_: 189 °C) nanocomposites are highly related to dispersibility of S-CNCs. However, after 4-dodecylbenzenesulfonic acid (DBSA) was added, the conductivity and thermal stability of SCNC/PANI increased up to 2.5 × 10^−1^ S cm^−1^ and 192 °C with almost no particle aggregation because of the increase in charge dispersion. The proposed biodegradable, renewable, and surface-modified S-CNC and C-CNC can be used in high-thermal-stability applications such as food packaging, optical films, reinforcement fillers, flexible semiconductors, and electromagnetic materials.

## Abstract 2

Biodegradable surface-modified CNCs were synthesized found high dispersibility and flexibility. Polyaniline-doped CNCs nanocomposites were exhibited high conductivity and thermal stability that may be promising for flexible semiconductors.

## Graphical abstract

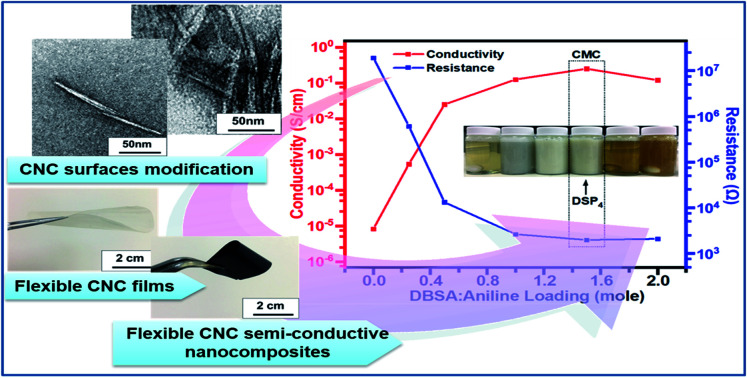

## Introduction

Cellulose nanocrystals (CNCs) have attracted considerable attention worldwide because of their high surface area, hydroxyl group-rich structure, nontoxicity, and high mechanical strength.^1,2^ Owing to their unique characteristics, CNCs are highly coveted for processes such as surface modification, drug delivery, film packaging, and pH detection.^3–6^ The dispersibility, thermal stability, and electrical conductivity of CNCs depend on surface charges, which prevent CNC aggregation in aqueous mediums due to electrostatic interaction.^7^ This interaction results in suspension particles. Therefore, grafting sulfate half-ester groups and carboxylic groups on CNC surfaces (S-CNC and C-CNC) increases CNC dispersibility. However, S-CNC results in lower thermal stability, dispersibility, and conductivity than C-CNC.

Intrinsically conductive polymers (ICPs) have been investigated for the development of supercapacitors. Among ICPs, polyaniline (PANI) has attracted considerable attention^8^ because of its highly tunable properties, easy and high-yield synthesis^9^ excellent environmental stability,^10^ and inexpensive monomers. PANI properties depend on factors such as the synthesis method, oxidant, dopant, and solution.^11^ High-conductivity (∼1.2 S cm^−1^) PANI can be synthesized in high yields (90%) through oxidative polymerization. Moreover, PANI prepared through emulsion polymerization has gained considerable attention because of its high solubility, solution processability, molecular weight, and electrical conductivity (24 S cm^−1^).^1,12^ However, PANI is brittle and insoluble in organic solvents, which results in poor processability and limits its application. Although researchers have focused on developing a suitable filler to modify PANI properties for use in devices such as corrosion inhibiter,^13^ supercapacitors,^14,15^ thermoelectric generators,^16,17^ and gas/vapor sensors,^18^ the degradation in mechanical performances of PANI-based composites due to the aggregation of particles and PANI brittleness remains a problem.^19^

Cellulose derivatives are ideal materials for reinforcing^20^ composites because of their renewability, sustainability, and biodegradability. For example, PANI lignocellulose composites are used as absorbents to remove commonly used dyes, such as congo red^21^ and eosin yellow,^22^ which are nonbiodegradable and poisonous to humans and the environment. The addition of nanocrystalline cellulose (NC) in the poly(lactic acid)/NC composites enhances their mechanical and hygroscopic properties and speeds up their degradation in soil. These changes are desirable when these components are used as food packaging materials.^4^ CNCs are used in electrospinning for the fabrication of advanced composites^23–27^ because of their favorable and flexible surface chemistry and high surface area, which enhance filtration performance and membrane stability for water purification applications^28^ as well as improve the thermal stabilities and mechanical properties of poly(lactic acid)/CNC nanocomposites.^29^ Polypyrene (PPy)/CNC supercapacitors outperform carbon nanotube and graphene supercapacitors.^30,31^ The conductivity of nanocomposite films PANI/microcrystalline cellulose (MCCs) synthesized through *in situ* polymerization increases with the gradual addition of aniline.^32^ Moreover, the addition of CNC drastically increases the conductivity of nanocomposite polyaniline/cellulose nanowhiskers/natural rubber.^33^ Thus, cellulose derivatives considerably improve nanocomposite performance. However, limited studies have been conducted on PANI-doped CNC surfaces, especially on how surface modification of CNC surfaces influences the performance of nanocomposites. Furthermore, though DBSA as surfactant and a protonating agent increased the electrical conductivity of polyaniline-doped DBSA;^34,35^ however, the limitation of DBSA concentration results of lower in conductivity due to the restricted electron delocalization. Moreover, how DBSA affect polyaniline-doped with different surface charge CNCs on conductivity, thermal properties, and morphology are not yet study comprehensively.

Therefore, we extracted CNCs from MCC by using through sulfuric acid hydrolysis and ammonium persulfate (APS) oxidation methods to grafted sulfated half ester groups (S-CNC) and carboxylic group on cellulose nanocrystals (C-CNC), respectively. Fourier transform infrared (FTIR) spectroscopy, thermogravimetric analysis (TGA), X-ray diffraction (XRD), dynamic light scattering (DLS), scanning electron microscopy (SEM), and transmission electron microscopy (TEM) were performed to characterize MCC, S-CNC, and C-CNC. We doped PANI on S-CNC and C-CNC through emulsion and *in situ* polymerization and compared the two methods in terms of the process time, difficulty of execution, and eco-friendliness. The properties of SCNC–PANI (SP), CCNC–PANI (CP), and DBSA–SCNC–PANI (DSP) nanocomposites were discussed by varying the PANI content, dopant ratios, surfactant ratios, and synthesis methods. The influence of S-CNC and C-CNC on the performance of PANI-based nanocomposites in terms of thermal properties, electrical conductivity, and surface roughness were investigated from a dispersion and colloidal stability perspective.

## Experimental

### Materials

Sulfuric acid (*M*_w_ = 98.072 g mol^−1^) was obtained from Scharlab, S.L, ammonium persulfate (APS, *M*_w_ = 228.18 g mol^−1^) was purchased from ADEKA Co., and 4-dodecylbenzenesulfonic acid (DBSA, *M*_w_ = 348.48 g mol^−1^) was obtained from ACROS Co. Aniline (ANI, *M*_w_ = 93.13 g mol^−1^) and aniline hydrochloride (*M*_w_ = 129.59 g mol^−1^) were purchased from Alfa Aesar. Hydrochloric acid (HCl, *M*_w_ = 36.46 g mol^−1^) and sodium hydroxide (NaOH, *M*_w_ = 40 g mol^−1^) were purchased from PanReac AppliChem. Uranyl acetate (*M*_w_ = 424.146 g mol^−1^) was purchased from Agar Scientific.

### Preparation of sulfate half ester onto cellulose nanocrystals (S-CNC)

**Fig. 1:**
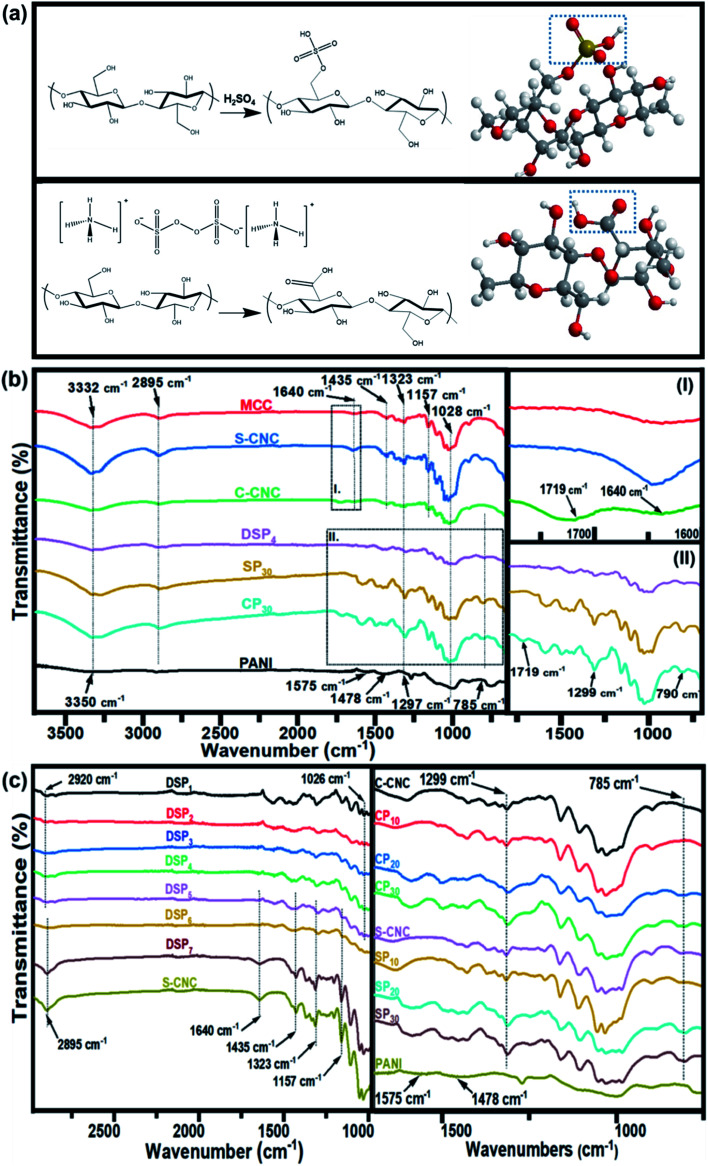
Synthesis process of (a) sulfated half-ester on cellulose nanocrystal (S-CNC) surfaces and carboxylic group on cellulose nanocrystal (C-CNC) surfaces. FTIR spectra of (b) MCC, S-CNC, C-CNC, DSP, SP, CP, and PANI. (c) DSP nanocomposites with various ANI/DBSA ratios and CNC/ANI ratios.

Sulfated grafted onto cellulose (S-CNC) was prepared by sulfuric acid hydrolysis^36,37^ of microcrystalline cellulose (MCC) as shown in Fig. 1a. MCC (39.59 g) was added in reactor with 188 mL water in an ice bath and stirred in high speed rate to prevent the heat, which may affect the reaction after adding the sulfuric acid. S-CNC hydrolysis was carried out with sulfuric acid (64% wt/wt) at 44 ± 1 °C with high speed stirring for 130 min. After hydrolysis, 500 mL of water (5 °C) was added to terminated the reaction followed by the centrifuging (Hermle Z383K, Tomy GRX-220) at the 12 000 rpm for 10 min until the supernatant turn into turbid appearance. After the centrifugation, the solids in suspension were collected and mixed with the water, then the homogeneous mixer (Shin Kwang Machinery HD-0025) was used to well-dispersed the bulk particles. The Ultrasonic Liquid Processors (Sonics & Materials, IncVCX750) were applied to sonicate the suspension in an ice bath for 30 min. Subsequently, the gel solution of S-CNC was investigated by the Dialysis Membrane (Orange ScientificOrDial D14 MWCO 12–14 kD) and kept in water for 7 days until solution achieved pH 5–6, then the powder of S-CNC was obtained by the freeze-drying process.

### Preparation of carboxylic groups onto cellulose nanocrystal (C-CNC)

Carboxylic groups onto cellulose nanocrystal (C-CNC) were prepared by the APS oxidation method.^38^ Firstly, 10 g of MCC was added in the reactor with the 1 L 1 M ammonium persulfate solution. After that, the temperature was increased to 60 °C with stirring rate 800 rpm for 16 hours until suspension became white haze looks, then the sample was centrifuged followed by the three times of repeated centrifuged and dialyzed to reached the solution conductivity to ∼5 μS cm^−1^ (pH 4). The powder of C-CNC was obtained by freeze-drying method and added in water dispersion system followed by the addition of sodium hydroxide until the pH value is neutrality to produce C-CNC in sodium form.

### Preparation of SCNC–PANI and CCNC–PANI nanocomposite films

The S-CNC and C-CNC were mixed with water separately, and sonicated in an ice bath with 40% for 10 min to obtained semi-transparent nanocrystal cellulose suspension (1.6 wt%). The 250 mL of suspension was stirred at 500 rpm followed by the addition of HCL–aniline. The mixture of APS (0.44 g)/water (20 mL) was added to the reactor for 24 h at room temperature under nitrogen condition. Once the reaction was completed, the solution was centrifugated at 8000 rpm for 10 min to remove the unreactive particle, then the solution of SCNC–PANI and CCNC–PANI was produced by sonicating in an ice bath for 10 min and coating on the Petri dish to fabricate the SCNC–PANI (SP) and CCNC–PANI (CP) nanocomposite films.

### Polymerization of SCNC–ANI by adding DBSA

The DBSA–SCNC–PANI (DSP) nanocomposites were prepared by adding DBSA *via* emulsion polymerization as shown in Fig. S6.†^39^ The mixture of S-CNC 0.94 g and water 74.6 mL was sonicated for 10 min in an ice bath to obtain the semi-transparent suspended liquid of nanocrystal cellulose. After that, the 0.46 mL aniline was first mixed with suspension (1.25 wt%) and stirred at 600 rpm, then the 3.08 mL (DBSA) was added in the mixture and stirred with high speed rate up to 800 rpm for 1 h. The DSP was mixed with the ammonium persulfate (APS) 1.14 g/water 25 mL and stirred vigorously under nitrogen flow condition for 24 h at room temperature. The centrifugation of suspension was done by centrifuging (Hermle Z383K, Tomy GRX-220) at 8000 rpm for 30 min to remove the by-product followed by the dialysis against deionized water, and the suspension was sonicated in an ice bath for 5 min to produce a well dispersed and homogenous of DSP. The suspension was dialyzed using Dialysis Membrane (Orange ScientificOrDial D14 MWCO 12 000–14 000 kD) and kept in the water for 1–2 days until the pH was approached to 6. As prepared DBSA/SCNC/PANI was dispersed in the 1 M HCl solution for 24 h at room temperature and casted on to the Petri dish to form the film. However, the addition of DBSA in C-CNC/PANI result of failure in film forming while casting.

## Measurements

### Fourier transform infrared (FTIR) spectroscopy

FTIR spectra of MCC, S-CNC, C-CNC, and nanocomposite films were investigated by the Thermo Scientific Nicolet iS50 at a resolution of 4 cm^−1^ with scanning range from 650 to 4000 cm^−1^ to analyze the characterization absorption of functional group.

### Dynamic light scattering (DLS)

The particle size distribution and zeta potentials (*ζ* potentials) of S-CNN, C-CNC, and DBSA–SCNC–PANI were obtained by Dynamic light scattering (DLS) using a Zetasizer Nano Series (Malvern Instruments Nano-ZS90). The particle size distribution was calculated by the mathematic equation.^40^ The zeta potentials were determined by the Smoluchowski equation, which *ζ* = *μη*/*ε*, where *μ*, *η*, *ε* are the electrophoretic mobility, viscosity, and dielectric constant, respectively. Various solutions were prepared and added into the PMMA tube, each sample was tested for three periods, each period was repeated measure for ten times to guarantee great reproducibility.

### Scanning electron microscope (SEM)

The surface morphology DSP, SP, and CP nanocomposite films were investigated by the Scanning Electron Microscope (SEM, JEOL JSM-6510). The samples were first gold-coated for 120 seconds for measurement at an accelerating voltage 10 kV.

### Field emission scanning electron microscope (FE-SEM)

The imaging of S-CNC and C-CNC were monitored by Field-Emission Scanning Electron Microscope (JEOL JSM-6330F), which provides higher magnification, higher resolution imaging, and lower potential risk of sample damage due to the fact of narrow electron beam with high electron energy.

### Transmission electron microscopy (TEM)

The TEM image of S-CNC and C-CNC were conducted by the transmission electron microscopy (Hitachi H-7650). The sample (∼100 μL) was prepared and dropped on the copper grid by micropipette followed by gettering. After drying at room temperature for 20 min, the negative staining techniques were applied by adding 100 μL uranyl acetate (UA), which left the unstained nanocellulose clear and more visible under black background.

### Four point probe

The resistance (*R*_c_) of nanocomposite films were measured by 4-point probing system (LRS4-TK1), which the resistivity (*ρ*) and electrical conductivity (*σ*) can be calculated as follow:*R*_c_ = *ρ*/*t*, *σ* = 1/*ρ**R*_c_: resistance. *ρ*: resistivity. *t*: thickness.

## Result and discussion

### Fourier transform infrared spectroscopy of cellulose nanocrystals

The Fourier transform infrared spectra of MCC, S-CNC, and C-CNC exhibited identical characteristic absorption peaks of cellulose (Fig. 1b) at approximately 3332, 2895, and 1640 cm^−1^, respectively, which corresponded to hydroxyl (OH) groups, the stretching vibration of C–H in the pyranoid ring, and the H–O–H bending of absorbed water in cellulose, respectively. The peaks at 1435, 1323, and 1157 cm^−1^ resulted from in-plane C–H bending, C–H deformation vibration, C=C stretching, and C–O–C stretching, respectively.^41^ However, an additional peak at 1719 cm^−1^ occurred in the spectra of C-CNC and CP_30_, which indicated that the hydroxyl groups of cellulose were converted into carboxyl groups on C-CNC surfaces during APS oxidation.^42^ Moreover, the sulfate half-ester on CNC surfaces at 1028 cm^−1^ (ref. 43) were negligible because of the overlapping period of twisting modes of the anhydroglucopyranose vibration unit (600–1800 cm^−1^).^44,45^

The characteristic peaks of PANI (Fig. 1b) were observed at 3350 (N–H stretching), 1575 (quinoid ring stretching vibration), and 1478 cm^−1^ (benzenoid rings stretching vibration).^46^ Additionally, the absorption peaks at approximately 1297, 1027, and 785 cm^−1^ are related to the stretching vibration of a secondary amine group, asymmetric SO_3_^−^ stretching, and the C–H bond (out-of-plane bending) of *para*-substituted benzene rings, respectively.^47,48^

For the spectra of DSP_4_, SP_30_, and CP_30_ nanocomposites (Fig. 1b), the peaks at approximately 785 (C–H bending) and 1299 cm^−1^ (C–N stretching) indicate a strong hydrogen bonding at the CNC/PANI interface. Subsequently, PANI was deposited on either S-CNC or C-CNC by either emulsion polymerization or *in situ* polymerization, respectively. The peaks at 1026 and 2920 cm^−1^ (Fig. 1c) became significant with the decrease in the ANI/DBSA from 4 to 0.67 (molar ratios). This phenomenon was attributed to the increase in the alkyl chain C–H vibration and asymmetric stretching of sulfonate groups of DBSA.^49^ Moreover, the characteristic absorption peaks at 2895, 1640, 1435, 1323, and 1157 cm^−1^ became apparent (Fig. 1c) with the increase in the CNC/ANI (mass ratios) from 1 to 5. Furthermore, intensify peaks were observed (Fig. 1c) at 785 (C–H bending) and 1299 cm^−1^ (C–N stretching) by enhancing the ANI concentration from 0% to 30% suggested the evidence of increase PANI content on the S-CNC and C-CNC surface.

### Aqueous dispersion and morphology of CNCs

**Fig. 2:**
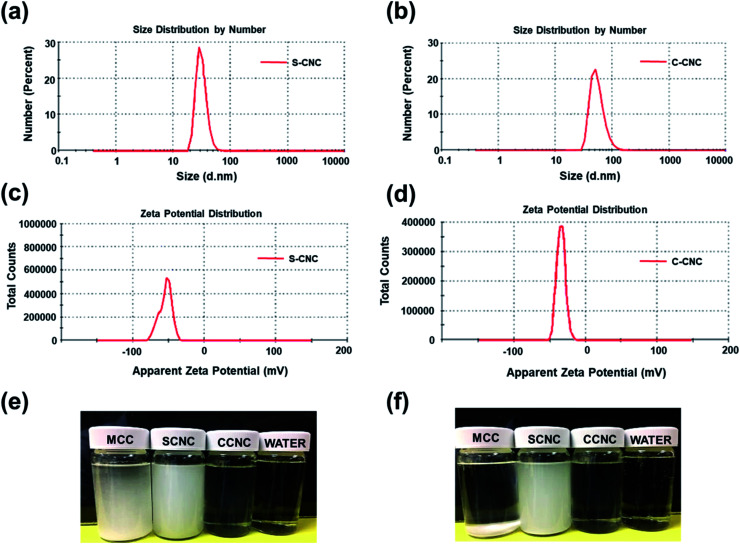
Particle size distribution of (a) S-CNC and (b) C-CNC (1% aqueous dispersion), zeta potential distribution of (c) S-CNC and (d) C-CNC (1% aqueous dispersion), particle sedimentation test of (e) MCC, S-CNC, and C-CNC, and water initially and (f) after 24 h.

**Fig. 3:**
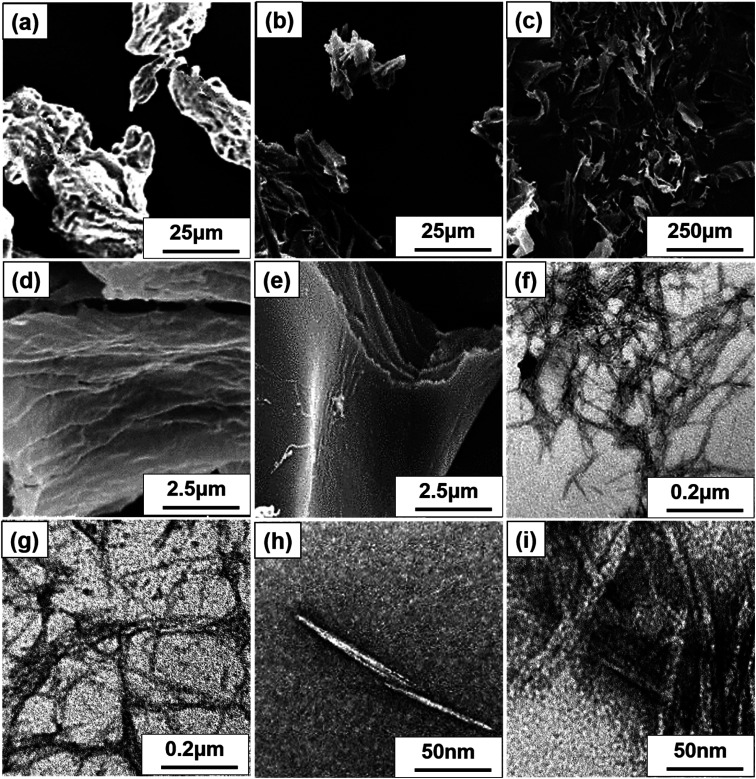
SEM image of (a) MCC, FE-SEM images of (b) S-CNC, and (c) C-CNC, SEM images of (d) S-CNC and (e) C-CNC. TEM images of (f) S-CNC and (g) C-CNC at 50 000 magnification, and (h) S-CNC and (i) C-CNC at 200 000 magnification.

SEM, FE-SEM, TEM, and DLS were performed to analyze the morphological images, particle size distribution, and zeta potential, respectively, of CNCs. Microscale particles (L: 20–100 μm; D: 20–100 μm) with the lowest aspect ratios are apparent in the SEM image of MCC (Fig. 3a). The lamellar (fibrous agglomerates) structure of S-CNC and C-CNC from the FE-SEM images (Fig. 3b and c) can be explained by the sublimation of ice molecules during the freeze-drying process when the cellulose particles were self-assembled into the ice lamellar system.^50^ The lamellae stacking of nanocellulose became apparent under high magnification (Fig. 3d and e). However, the single cellulose nanocrystal cannot be measured through either SEM or FE-SEM. TEM revealed the rod-like crystals of S-CNC (L: 150–400 nm; D: 10–20 nm) and C-CNC (L: 100–200 nm; D: ∼10 nm) [Fig. 3f and g, respectively]. In particular, the negative-staining electron microscopy conducted using uranyl acetate (UA) combined with carbon grids revealed dark backgrounds as well as a more distinct TEM images of cellulose nanocrystals (Fig. S1†). However, the TEM image provided a limited view of the specimen, and it could not represent the correct morphology of cellulose nanocrystals. Therefore, the particle size distribution of S-CNC and C-CNC were investigated through DLS to ascertain the particle size. The particle size distribution of cellulose nanocrystals ranged from 30 to 200 nm, and the average particle sizes of S-CNC (191.2 nm) and C-CNC (126.2 nm) were determined (Fig. 2a and b). TEM and DLS revealed that S-CNC exhibited the largest particle size as well as the highest aspect ratios (Table 1). The mechanical properties of nanocomposites can considerably improve with the minimum loading of S-CNC. The negative charge of the S-CNC surface (R–OSO_3_^−^) was confirmed through the zeta potential value of −54.7 mV. The zeta potential of C-CNC was −34.3 mV. The zeta potential of both S-CNC and C-CNC were higher than −30 mV, suggesting a stable nanocellulose suspension.^51^ However, S-CNC exhibited a higher zeta potential than did C-CNC, which suggested highly stable particle suspension with superior capability to hinder nanoparticle agglomeration typically requires a zeta potential greater than −35 mV.^52^ The repulsive force was stronger than the van der Waals force of attraction in S-CNC suspensions, which indicated a higher dispersibility of S-CNC than that of C-CNC. Thus, S-CNC exhibited considerable film formability during casting as well as high surface area in contact with the polymer matrix, ensuring the resulting nanocomposites are of a high quality.^53^ By contrast, C-CNC exhibited a poor film formability when casting, and the particles aggregated easily, which resulted in a brittle structure with a white appearance (Fig. S2b†). Particle sedimentation tests of C-CNC suspensions were repeated several times to ensure their reproducibility. The nontransparent with partial sediment of a C-CNC suspension were observed after sonication (Fig. 2e and f). The carboxylic groups on C-CNC were highly hydrophilic and formed hydrogen bonds in water, which resulted in considerable stiffness in films. However, C-CNC (electrostatic interactions) aggregation resulted in film casting failure with frangible surface. As we prepared the 2 wt% (transparent) and 5 wt% (gel-like) C-CNC–COONa^+^ aqueous suspensions by adding NaOH, a new film with certain stiffness and flexibility was formed by casting (Fig. S2b†). The zero value of zeta potential of the C-CNC–COONa^+^ suspension indicated it had the lowest stability (*i.e.*, maximum coagulation) and highest solubility.^54^ Therefore, the C-CNC film was successfully fabricated by improving aqueous solubility of C-CNC.

**Table 1:** Properties of microcrystalline cellulose (MCC), cellulose nanocrystals (S-CNC, C-CNC)

Filler	Length	Diameter	Aspect ratios	Psd, nm	Zeta *P*, mV	*T*_0_	pH
MCC	20–100 μm	20–100 μm	1.8–6.4			320	
S-CNC	150–400 nm	10–20 nm	18	191.2	−54.7	237; 168	5–5.5
C-CNC	100–200 nm	∼10 nm	15	126.2	−34	211	7

### Morphology of nanocomposite films

**Fig. 4:**
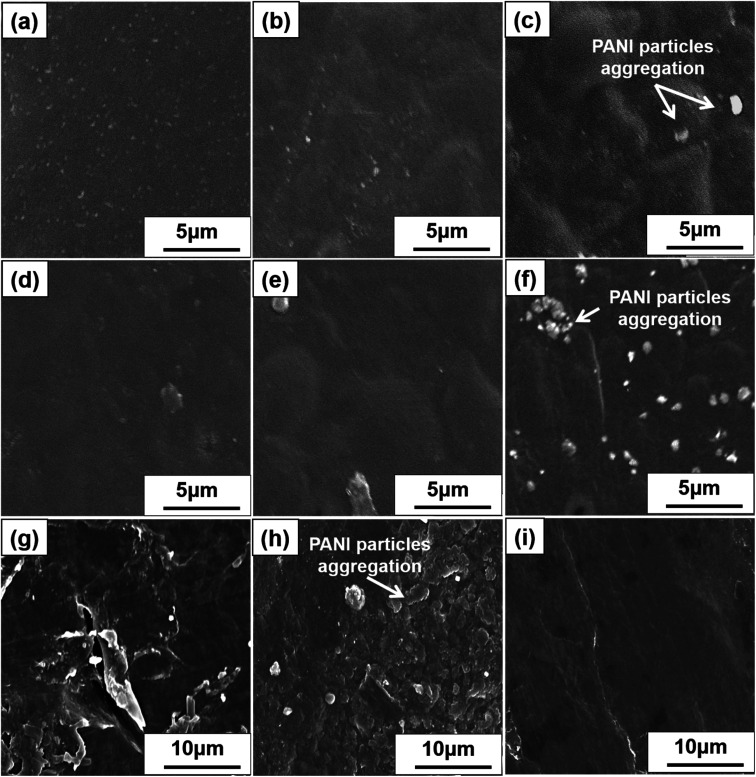
SEM images of SP nanocomposites containing PANI (a) 10%, (b) 20%, and (c) 30% and CP nanocomposites containing PANI (d) 10%, (e) 20%, (f) and 30%. (g) PANI and (h) DSP nanocomposites with ANI/DBSA ratios of 4 : 1 and (i) 2 : 3 (molar ratios).

Fig. 4a, d and g displays the SEM images of SP nanocomposites containing 10, 20, and 30 wt% PANI, respectively. Smooth and well-dispersed surfaces were observed at 10 and 20 wt%, but a few PANI particles were observed in Fig. 4g. Fig. 4b, e and h displays the SEM images of CP nanocomposites with 10, 20, and 30 wt% PANI, respectively. Both SP and CP nanocomposites shows apparent PANI particle aggregation with an increase in PANI content. However, SP nanocomposites exhibited limited PANI particle aggregation compared with CP because the high dispersibility of S-CNC resulted in a large surface area in contact with PANI. Therefore, PANI particle dispersion on S-CNC surfaces was higher than that on the C-CNC surface. Fig. 4c displays the SEM image of PANI, and Fig. 4f and i display DSP nanocomposites with ANI/DBSA = 4 and 0.67 (molar ratios), respectively. SEM revealed that with the decrease in the ANI/DBSA, resulting in it having a smooth surface (Fig. 4i), which suggest DBSA concentration was over the critical micelle concentration, and the micelle form. However, the low concentration of DBSA resulted in a rough surface with considerable aggregation of PANI particles (Fig. 4f). Notably, in contrast to SP_30_ nanocomposites (Fig. 4g) and CP_30_ (Fig. 4h), in which nano PANI particles (0.5–1.7 μm) were observed, the DSP_4_ surface (Fig. 4i) exhibited uniform morphology without particle aggregation.

### Thermal behavior of S-CNC, C-CNC, and nanocomposite films

**Fig. 5:**
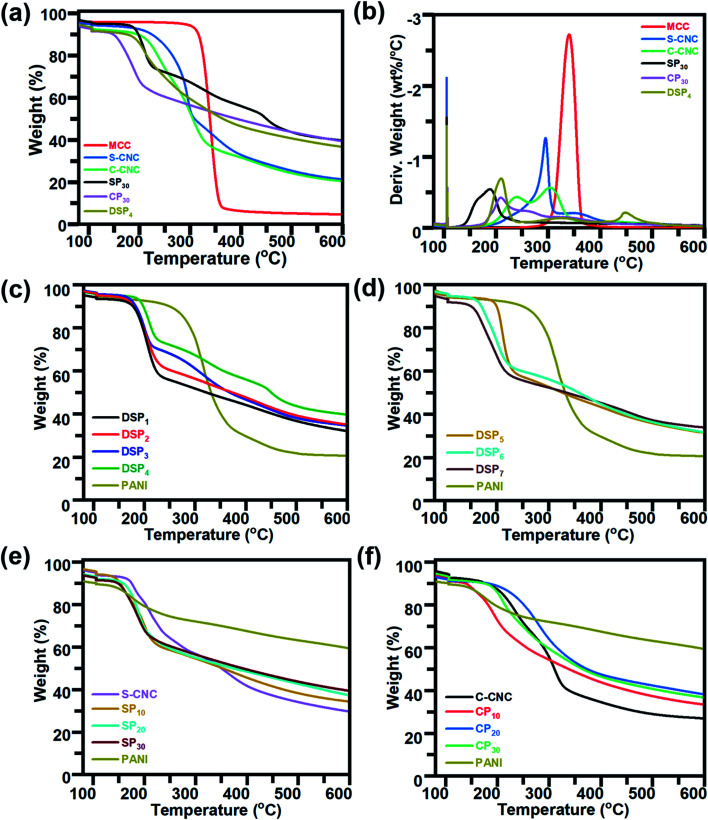
TGA and DTG curves of (a and b) MCC, S-CNC, C-CNC, and nanocomposites (DSP, SP, and CP) containing 30wt% PANI. TGA curves of (c) nanocomposites DSP_1_, DSP_2_, DSP_3_, and DSP_4_, (d) DSP_5_, DSP_6_, and DSP_7_, (e) S-CNC, SP containing PANI (10%, 20%, and 30%), and PANI, (f) C-CNC, CP containing PANI (10%, 20%, and 30%), and PANI.

The thermal properties of MCC, S-CNC, and C-CNC, nanocomposites (DSP, SP, CP) were investigated through TGA and DTG curves (Fig. 5a). The DTG curves of all samples displayed peaks at approximately 100 °C, which indicated water evaporation. The maximum weight loss temperatures (*T*_max_) of MCC, S-CNC, and C-CNC (Fig. 5b) were 294, 305, and 339 °C, respectively, which was attributed to the degradation of cellulose pyrolysis.^55^ The lower thermal stabilities of nanosized S-CNC and C-CNC than micro-sized MCC were attributed to the increase in the surface area subjected to heat treatment. Thus, a high decomposition rate was observed.^56,57^ Furthermore, the replacement of hydroxyl groups by the sulfate half-easter groups (–SO_3_H) during sulfuric acid hydrolysis reduced the activation energy, which expedited decomposition.^58,59^ The introduction of carboxylic groups (–COOH) during the APS oxidation process on the C-CNC surface resulted in unsteady anhydroglucuronate units, which contributed to the earlier weight degradation of C-CNC than that with MCC and S-CNC.^60^

DSP_4_, SP_30_, and CP_30_ nanocomposites (Fig. 5a) decomposed at even lower temperatures than S-CNC and C-CNC did. However, these films exhibited superior thermal stabilities over nanocelluloses in the temperature range 280–600 °C with a limited 20% weight loss. For aniline content fixed at 30 wt%, for different polymerization methods, DSP_4_ (*T*_0_: 192 °C, *T*_max_: 209 °C) resulted in a higher thermal stability than SP_30_ did (*T*_0_: 156.8 °C, *T*_max_: 187.6 °C), but a tipping point was observed at 215 °C. With various nanocelluloses (S-CNC, C-CNC) involved in the *in situ* polymerization system, CP_30_ (*T*_0_: 189.9 °C, *T*_max_: 209.4 °C) exhibited a higher thermal stability than SP_30_ (*T*_0_: 156.8 °C, *T*_max_: 187.6 °C) did. However, thermal stability decreased from 337.6 to 600 °C.

In the DTG curves, the peaks from 25 to 120 °C (Fig. S4a†) represented the degradation of volatiles and absorption water from DSP. Three major peaks (A, B, C) were observed. Peak A (120–220 °C), with a maximum degradation temperature range of 205–310 °C, represented the weight loss of cellulose nanocrystals.^61^ B peaks at 315 °C associated with the interaction between the PANI chain with DBSA (NH^+^…SO^3^),^62^ and the degradation of oxidation of PANI and surplus DBSA started at 300 °C.^63^ The C peaks at 425 °C contributed to the decomposition of the PANI backbone.^63^ The thermal stability of DSP nanocomposites improved with a decrease in the ratios of ANI/DBSA from 4 to 0.67 (molar ratios) (Table S1†) (Fig. 5c). DSP_4_ exhibited the highest onset temperature at 192 °C and lowest weight loss at 600 °C (residual mass: 40%). The decline in the ANI/CNC ratio from 1 to 0.2 (mass ratios) increased the thermal stability of DSP nanocomposites (Fig. 5d). DSP_1_ exhibited the highest onset temperature of 199.9 °C.

The SP_20_ nanocomposite was thermally more stable (*T*_0_: 167.5 °C) than SP_10_ (*T*_0_: 144.9 °C) and SP_30_ (*T*_0_: 156.8 °C) were; SP_10_ exhibited the lowest onset temperature (Fig. 5e) because excess S-CNC resulted in self-condensation, which affected the SCNC–PANI interaction as well as thermal stability.^64^ However, SP_30_ exhibited the highest thermal stability at higher temperature range with maximum residual mass (40%). At temperature above 300 °C, all the nanocomposite films remained more residual mass than S-CNC, C-CNC, and MCC because the addition of rigid π-conjugated backbone molecular structure of PANI.^1^ Excess concentrations of either C-CNC or PANI resulted in lower thermal stabilities of CP_10_ and CP_30_ nanocomposites (Table S1†) because of the self-agglomeration of C-CNC and the influence of PANI on the C-CNC–PANI interface (Fig. 5f). Both SP and CP nanocomposites exhibited the best thermal stability at 20 wt% PANI. Of the samples, the CP_20_ nanocomposite exhibited the highest thermal stability (*T*_0_: 224.0 °C; Table S1†).

The CP nanocomposites exhibited higher thermal stability than SP nanocomposites did, which can be explained by C-CNC particle agglomeration during polymerization (low zeta potential and poor dispersibility). The DSP, SP, and CP nanocomposites exhibited lower thermal stability than S-CNC and C-CNC did, which can be explained by the reduced inter- and intra-molecular interaction of H-CNC on the surface of PANI-doped CNC.^48^ However, the higher thermal stability than PANI films because crystalline nanocellulose merged into PANI, which resulted in the expansion of the PANI crystalline structure. Thus, high energy was required to decompose water/acid from the polymer chain.^65,66^

### Electrical properties of nanocomposite films

**Fig. 6:**
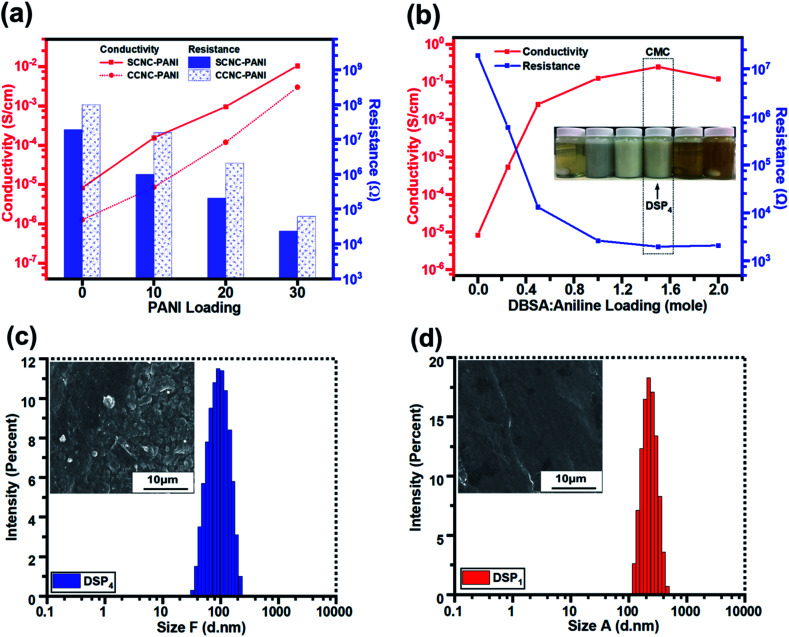
The conductivity and resistance (a) of DSP nanocomposites corresponding to DBSA/ANI loading, (b) of SP and CP nanocomposites against PANI loading. The particle size distribution and SEM images of (c) DSP_4_ and (d) DSP_1_.

Fig. 6a illustrates that the electrical conductivities of S-CNC and C-CNC films were poor (8.26 × 10^−6^ and 1.27 × 10^−6^ S cm^−1^), respectively (Table 2). However, C-CNC and S-CNC exhibited highly improved conductivity after PANI doping, with SP_30_ (1.06 × 10^−2^ S cm^−1^) and CP_30_ (3.0 × 10^−3^ S cm^−1^) exhibiting the highest conductivity with the lowest resistance (2.35 × 10^4^ and 6.22 × 10^4^ Ω) were observed from using PANI at 30 wt%. Both SP and CP nanocomposites exhibited an increase in conductivity with a decrease in resistance when the CNC/ANI–HCL ratio was decreased. However, SP exhibited a higher electrical conductivity than CP did for every ratio (Fig. 6a), which can be explained by a high stable suspension of S-CNC and a higher capability to hinder CNC particle agglomeration.^52^

**Table 2:** Composition and properties of SCNC–PANI and CCNC–PANI prepared by *in situ* polymerization

	PANI (wt%)	ANI–HCL : CNC (wt%)	Aqueous CNC (wt%)	Conductivity (S cm^−1^)	Resistance (Ω)
S-CNC	0	0		8.26 × 10^−6^	1.92 × 10^7^
SP_10_	10	1 : 9		1.55 × 10^−4^	9.88 × 10^5^
SP_20_	20	2 : 8		9.60 × 10^−4^	2.07 × 10^5^
SP_30_	30	3 : 7		1.06 × 10^−2^	2.35 × 10^4^
C-CNC	0	0		1.27 × 10^−6^	9.8 × 10^7^
CP_10_	10	1 : 9		8.60 × 10^−6^	1.56 × 10^7^
CP_20_	20	2 : 8		1.19 × 10^−4^	2.09 × 10^5^
CP_30_	30	3 : 7		3.00 × 10^−3^	6.22 × 10^4^

The conductivity and resistance of DSP nanocomposites were monitored by decreasing the ANI/DBSA (molar ratios) as supported by the critical micelle concentration (CMC), as displayed in Fig. 6b. The conductivity of DSP_1_ was poor (5.4 × 10^−4^ S cm^−1^) with limited DBSA (surfactant) exhibited considerable particle sedimentation in the suspension and large particle size obtained by DLS (Fig. S3c†). However, the increase in the DBSA/ANI ratio from 0.5 to 4 (molar ratios) enhanced conductivity from 5.4 × 10^−4^ to 2.5 × 10^−1^ S cm^−1^ (three orders of magnitude higher) (Table S1†) and reduced the particle size from 250 to 100 nm (Fig. S3b†). Because solute solubility in the micelle solution affects the size of micelles and their structure,^67^ the DSP_4_ suspension with a high DBSA concentration exhibited no particle sedimentation (Fig. S3a†). The lowest particle sizes (Fig. S3d†) suggested that maximum solutes were dissolved in the micelle solution of DSP_4_. DSP_1_ displays obvious particle sedimentation with the largest particle size (Fig. S3a†), which eventually resulted in rough surfaces on SEM images (Fig. 6f and i) and low conductivity. Notably, DSP_3_ (1.25 × 10^−1^ S cm^−1^) and DSP_4_ (2.5 × 10^−1^ S cm^−1^) nanocomposite films had the same order of magnitude as the PANI film with sufficient DBSA to pass the CMC (Table 3). The DSP_4_ nanocomposites (2.5 × 10^−1^ S cm^−1^) exhibited conductivity one order of magnitude higher than that of the SP_30_ nanocomposites (1.06 × 10^−2^ S cm^−1^) because of the DBSA (surfactant) electrostatic interaction of negative charged CNCs.

**Table 3:** The composition of DBSA–SCNC–PANI prepared by emulsion polymerization

	ANI : CNC (mass)	ANI : DBSA (molar)	APS : ANI (molar)	Conductivity (S cm^−1^)	Resistance (Ω)
DSP_1_	0.5	4	1	5.4 × 10^−4^	6.12 × 10^5^
DSP_2_	0.5	2	1	2.5 × 10^−2^	1.33 × 10^4^
DSP_3_	0.5	1	1	1.25 × 10^−1^	2.65 × 10^3^
DSP_4_	0.5	0.67	1	2.5 × 10^−1^	1.99 × 10^3^
DSP_5_	0.5	0.5	1	1.9 × 10^−2^	3.44 × 10^3^
PANI	0	1	1	1.19 × 10^−1^	2.10 × 10^3^
DSP_6_	1	0.5	1	2.9 × 10^−2^	1.14 × 10^4^
DSP_7_	0.2	0.5	1	1.17 × 10^−2^	2.13 × 10^3^

**Fig. 7:**
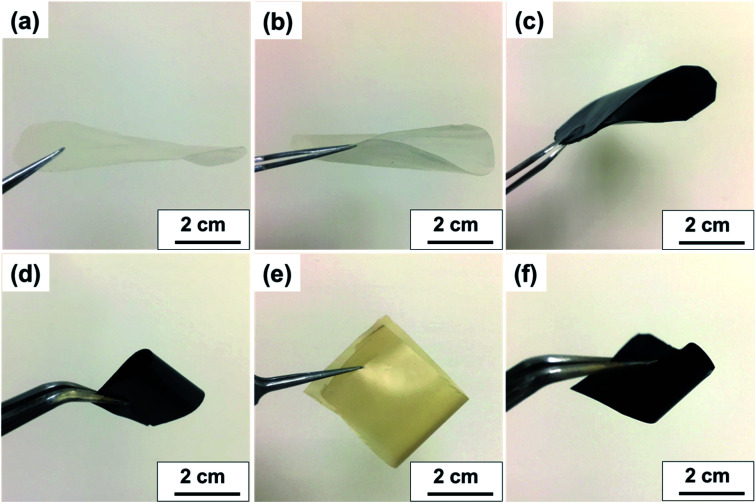
Visual aspect of (a) flat S-CNC films, (b) flexible S-CNC films bent by 180°, (c) SP_4_ nanocomposite, (d) SP_30_ nanocomposite, (e) flexible C-CNC films bent by 180°, (f) CP_30_ nanocomposite.

Fig. 7a and b displays the transparency of S-CNC films in the original state and the S-CNC bent by 180°, respectively. The smooth and well-dispersed DSP_4_ nanocomposites (Fig. 7c) and SP_30_ nanocomposites (Fig. 7d) retained the flexibility of S-CNC films after PANI doping (dark appearance). The C-CNC film exhibited a light-yellow color and high flexibility (bent 180°), and the homogenous CP_30_ nanocomposites retained the flexibility of the C-CNC film. The major difference between nanocomposites prepared through emulsion and *in situ* polymerization of rough surfaces are displayed in Fig. S5a and b.† The DSP films had smooth surface compare with rough surface of SP films with some spherical bulge. Moreover, the SP films (Fig. S5b†) exhibited limited particle aggregation compared with CP films (Fig. S5b†) because of the stable suspension of S-CNC.

## Conclusion

CNCs dispersibility were successfully increased by grafting sulfate half-ester and carboxylic groups. This increase in dispersibility resulted in higher charge S-CNC and C-CNC suspension. The influence of the preparation methods (sulfuric acid hydrolysis and APS oxidation methods) on the morphology, dispersibility, thermal stability, and conductivity of pure S-CNC and C-CNC films were investigated. Of the two methods, the APS oxidation method was less time consuming, easier to execute, and resulted in higher yields (∼60–35%). However, the S-CNC prepared using the sulfuric acid hydrolysis method exhibited high aspect ratios (∼18; 15), greater thermal stability (*T*_0_: 237 °C; 211 °C), higher dispersibility (−54 mV; −34 mV), higher electric conductivity (8.3 × 10^−6^ S cm^−1^; 1.2 × 10^−6^ S cm^−1^), and superior film formability. The low dispersibility of C-CNC resulted in poor film formability. Enhancing the solubility of C-CNC resulted in improved film formability. However, the addition of DBSA decreased the solubility of C-CNC/PANI prepared through emulsion polymerization, which resulted in a broken film with large particle aggregation (Fig. S7a and b†). Flexible, biodegradable, and semiconductive DSP, CP, and SP nanocomposites were successfully fabricated. The DSP_4_ nanocomposite exhibited the highest electrical conductivity (2.5 × 10^−1^ S cm^−1^), high thermal stability (*T*_0_: 192 °C), and a well-dispersed surface when the DSP suspension had sufficient DBSA to form micelles and over the critical micellar concentrations (CMC) while the smallest particle size of DSP_4_ suspensions also indicated the maximum number of solutes were dissolved in the micelle solution for polymerization to produce quality nanocomposites. Furthermore, the ratios of nanofiller and surfactant affected the conductivity and thermal stability of DSP. SP nanocomposites exhibited higher conductivity, lower thermal stability, and better morphology than CP nanocomposites did due to instability of the C-CNC suspension. The conductivity of SP and CP increased with an increase in PANI concentration. However, both SP_20_ and CP_20_ exhibited thermal stability. Under an identical PANI (30 wt%) concentration, the DSP_4_ nanocomposite exhibited a higher electrical conductivity (2.5 × 10^−1^ S cm^−1^), thermal stability (*T*_0_: 192 °C), and smoother surface than SP_30_ and CP_30_ did because of a higher charge DSP suspension resulting from the addition of DBSA. Dispersibility of S-CNC and C-CNC suspension considerably affects the properties of pure CNCs and nanocomposite films, and S-CNC is more valuable than C-CNC. Both emulsion and *in situ* polymerization are ecological, safe, and economical. Although emulsion polymerization is time consuming and complex, it produced high quality nanocomposites with higher conductivity, greater thermal stability, and smoother surfaces than *in situ* polymerization did.

## Conflicts of interest

There are no conflicts to declare.

## Supplementary Material

RA-011-D0RA10663A-s001
