# Supplementary material for: Enhanced electrical and thermal properties of semi-conductive PANI-CNCs with surface modified CNCs
Source: RSC Adv. 2021 Mar 19;11(19):11444–56. doi: 10.1039/d0ra10663a (PMC8695952; doi:10.1039/d0ra10663a)
Supplement: RA-011-D0RA10663A-s001 [file RA-011-D0RA10663A-s001.pdf]

Electronic Supplementary Material (ESI) for RSC Advances.
This journal is © The Royal Society of Chemistry 2021

Supporting Information

Enhanced electrical and thermal properties of semi-conductive PANI-CNCs with surface modified CNCs

Po-Yun Chen,[a,+] Chieh Hsu,[a,+] Manikandan Venkatesan,[a] Yen-Lin Tseng,[a] Chia-Jung Cho,[a,*] Su-Ting Han,[b] Ye Zhou,[c] Wei-Hung Chiang,[d] and Chi-Ching Kuo,[a,*]

[a] Institute of Organic and Polymeric Materials, Research and Development Center of Smart Textile Technology, National Taipei University of Technology, Taipei 10608, Taiwan

*Author to whom all correspondence should be addressed

Tel: 886-2-27712171*2407 Fax: 886-2-27317174

Correspondence to: *Prof. C.-C. Kuo: kuocc@mail.ntut.edu.tw

*Dr. C.-J. Cho: ppaul28865@mail.ntut.edu.tw

Table S1. Thermal properties and conductivity of MCC, S-CNC, C-CNC, SCNC-PANI, CCNC-PANI, and DBSA-SCNC-PANI nanocomposites.

Sample	C (S/cm)	T_o (°C)	T_{max} (°C)	$T_{5\%}$ (°C)	$T_{15\%}$ (°C)	Residual mass (%)
MCC	N/A	320	340.1	285.0	321.9	4.7
S-CNC	8.26×10^{-6}	237	294.8	99.2	249.0	21.3
C-CNC	1.27×10^{-6}	211	304.5	69.0	222.9	20.5
SP_{10}	1.55×10^{-4}	145.0	199.6	105.3	164.9	34.4
SP_{20}	9.60×10^{-4}	167.5	188.7	68.1	174.3	37.3
SP_{30}	1.06×10^{-2}	156.8	187.6	63.4	165.2	40.0
CP_{10}	8.60×10^{-6}	158.4	192.6	64.8	164.1	33.4
CP_{20}	1.19×10^{-4}	225.6	277.9	54.3	228.5	38.2
CP_{30}	3.00×10^{-3}	189.9	209.4	75.2	202.0	36.7
DSP_1	5.4×10^{-4}	177.3	206.7	121.4	189.7	35.06
DSP_2	2.5×10^{-2}	179.3	199.5	153.2	193	34.7
DSP_3	1.25×10^{-1}	182.4	182.8	84.8	189.5	32.1
DSP_4	2.5×10^{-1}	192.0	209.6	148.7	206.2	40.0
DSP_5	1.9×10^{-2}	168.8	211	105.6	180.1	31.64
DSP_6	2.9×10^{-2}	199.9	210	102.5	205.6	31.49
DSP_7	1.17×10^{-2}	156.2	195	78.1	166.1	33.9

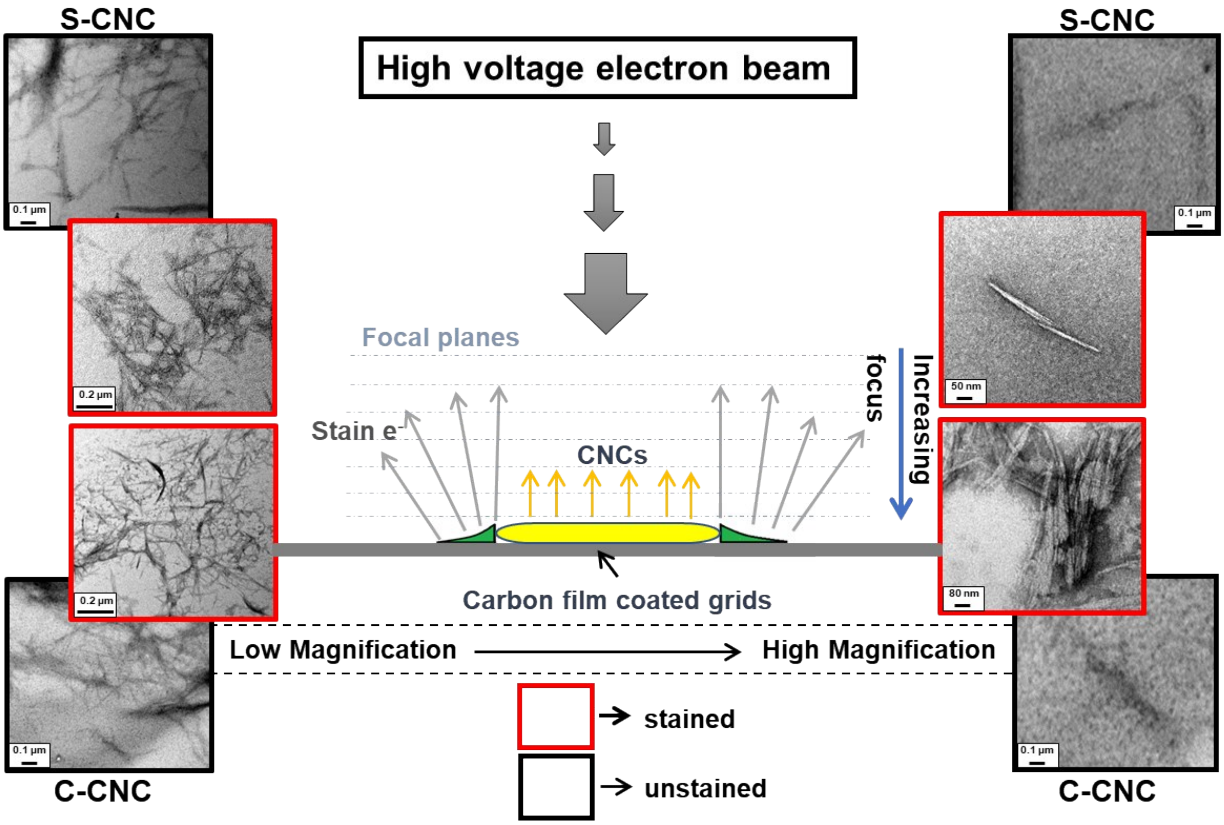

Figure S1. Comparison between negative stain electron microscopy (Red) by using Uranyl acetate (UA) combined with carbon grid exhibited higher resolution under dark background and transmission electron microscopy (Black)

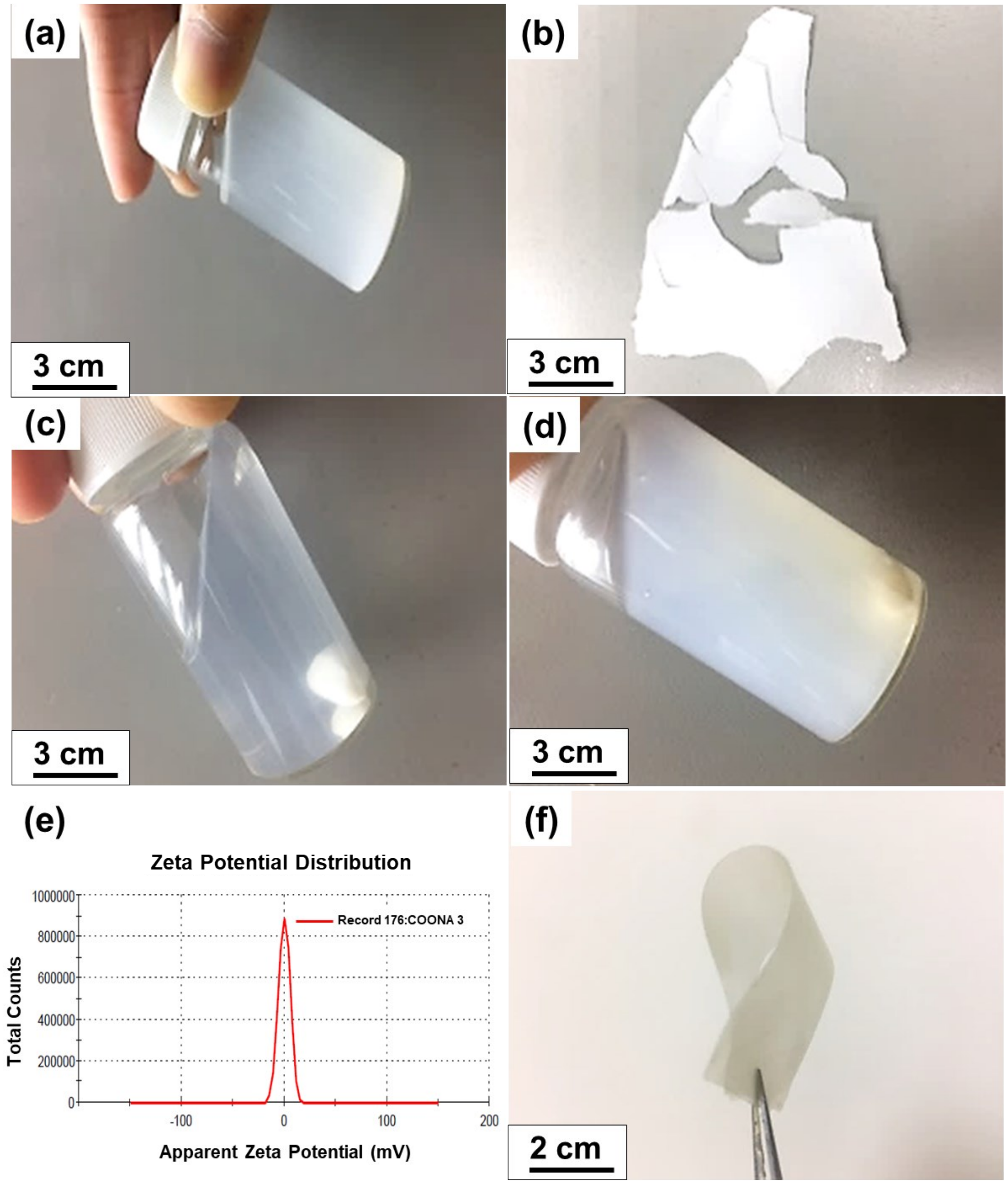

Figure S2. Visual aspect of (a) C-CNC aqueous, (b) C-CNC films before modification, (c) 2wt% C-CNC-$COONa^{+}$ aqueous, (d) 5wt% C-CNC-$COONa^{+}$ aqueous, (e) Zeta potential distribution of 5wt% C-CNC-$COONa^{+}$, (f) C-CNC films after modification.

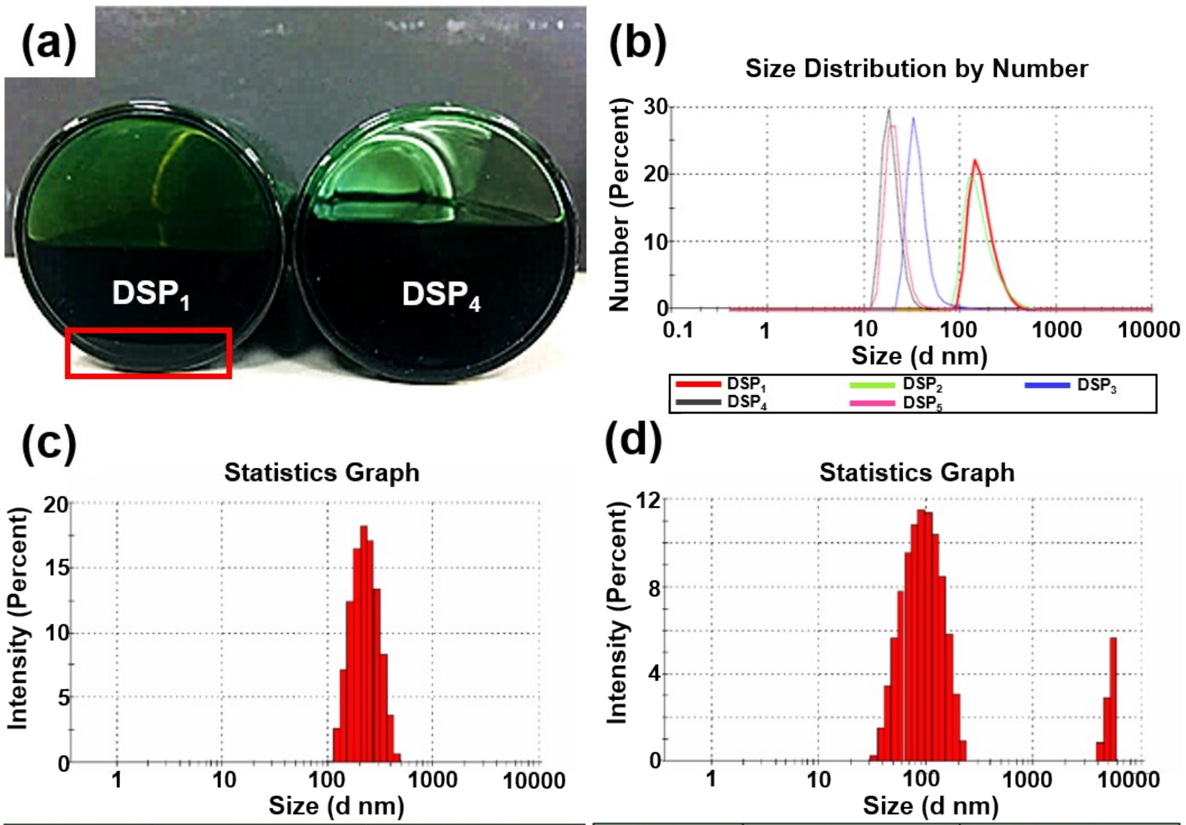

Size d.nm	Mean Intensity Percent	Std Dev Intensity Percent
78.82	0.0	
91.28	0.0	
105.7	0.1	
122.4	2.6	
141.8	7.1	
164.2	12.3	
190.1	16.5	
220.2	18.3	
255.0	17.1	
295.3	13.4	
342.0	8.3	
396.1	3.6	
458.7	0.7	
531.2	0.0	
615.1	0.0	
712.4	0.0	
825.0	0.0	
955.4	0.0	

Size d.nm	Mean Intensity Percent	Std Dev Intensity Percent
78.82	10.8	
91.28	11.5	
105.7	11.4	
122.4	10.4	
141.8	8.4	
164.2	5.8	
190.1	3.1	
220.2	1.0	
255.0	0.0	
295.3	0.0	
342.0	0.0	
396.1	0.0	
458.7	0.0	
531.2	0.0	
615.1	0.0	
712.4	0.0	
825.0	0.0	
955.4	0.0	

Figure S3. Settling test of DSP$_1$ and DSP$_4$ suspension after emulsion polymerization, (b) Particle size distribution of DSP$_1$, DSP$_2$, DSP$_3$, DSP$_4$, and DSP$_5$, (c) Particle size distribution of DSP$_1$ and DSP$_4$.

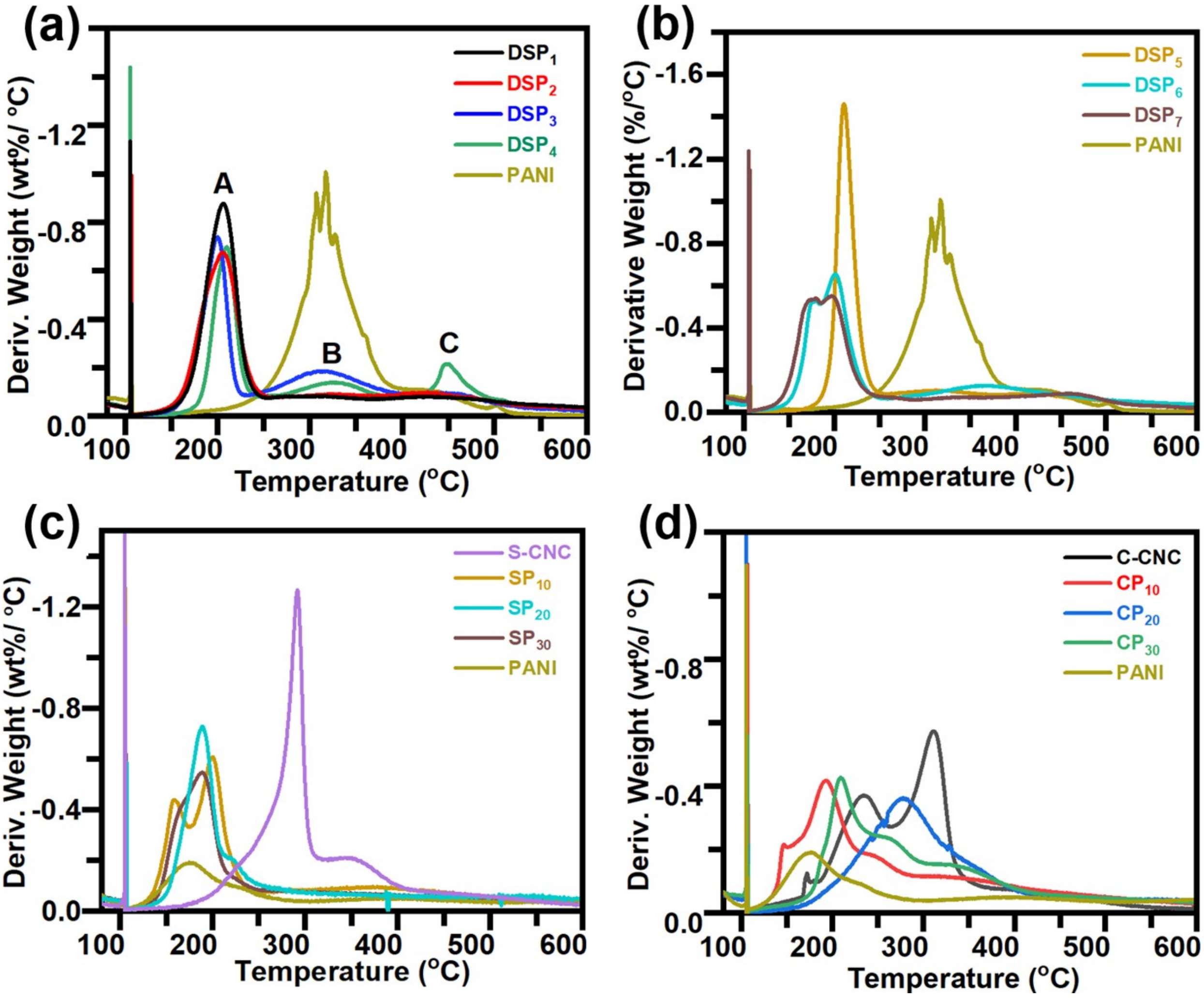

Figure S4. DTG curves of (a) DSP_1, DSP_2, DSP_3, DSP_4, and PANI, (b) DSP_5, DSP_6, DSP_7, and PANI, (c) S-CNC, SP_{10}, SP_{20}, SP_{30}, and PANI, (d) C-CNC, CP_{10}, CP_{20}, CP_{30}, and PANI.

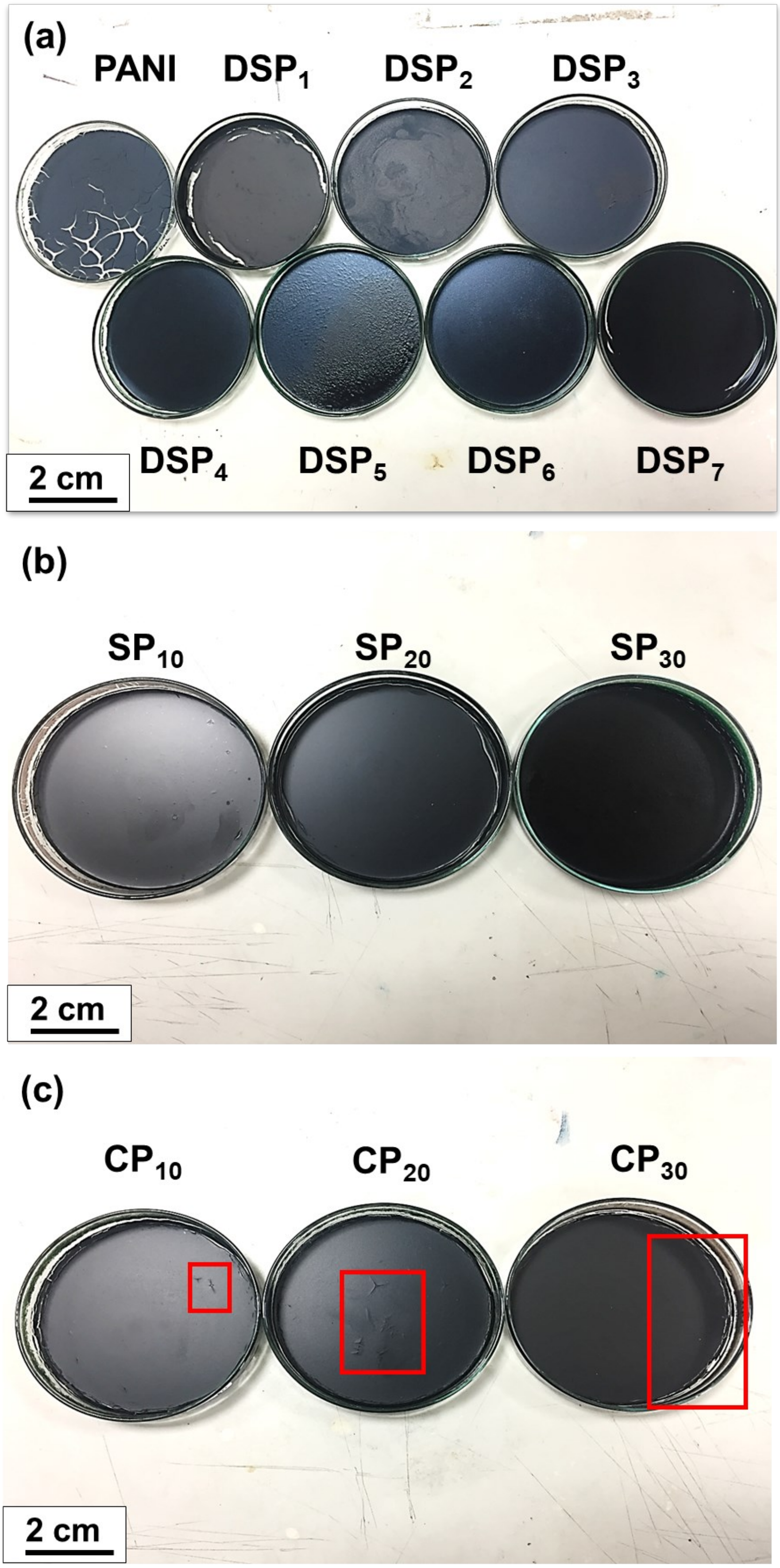

(a)
PANI
DSP1
DSP2
DSP3
DSP4
DSP5
DSP6
DSP7
2 cm
(b)
SP10
SP20
SP30
2 cm
(c)
CP10
CP20
CP30
2 cm

Figure S5. Photographs of (a) PANI, DSP_1, DSP_2, DSP_3, DSP_4, DSP_5, DSP_6, DSP_7 films casting on the petri deck, (b) SP_{10}, SP_{20}, SP_{30} films casting on the petri deck, (c) CP_{10}, CP_{20}, CP_{30} films casting on the petri deck.

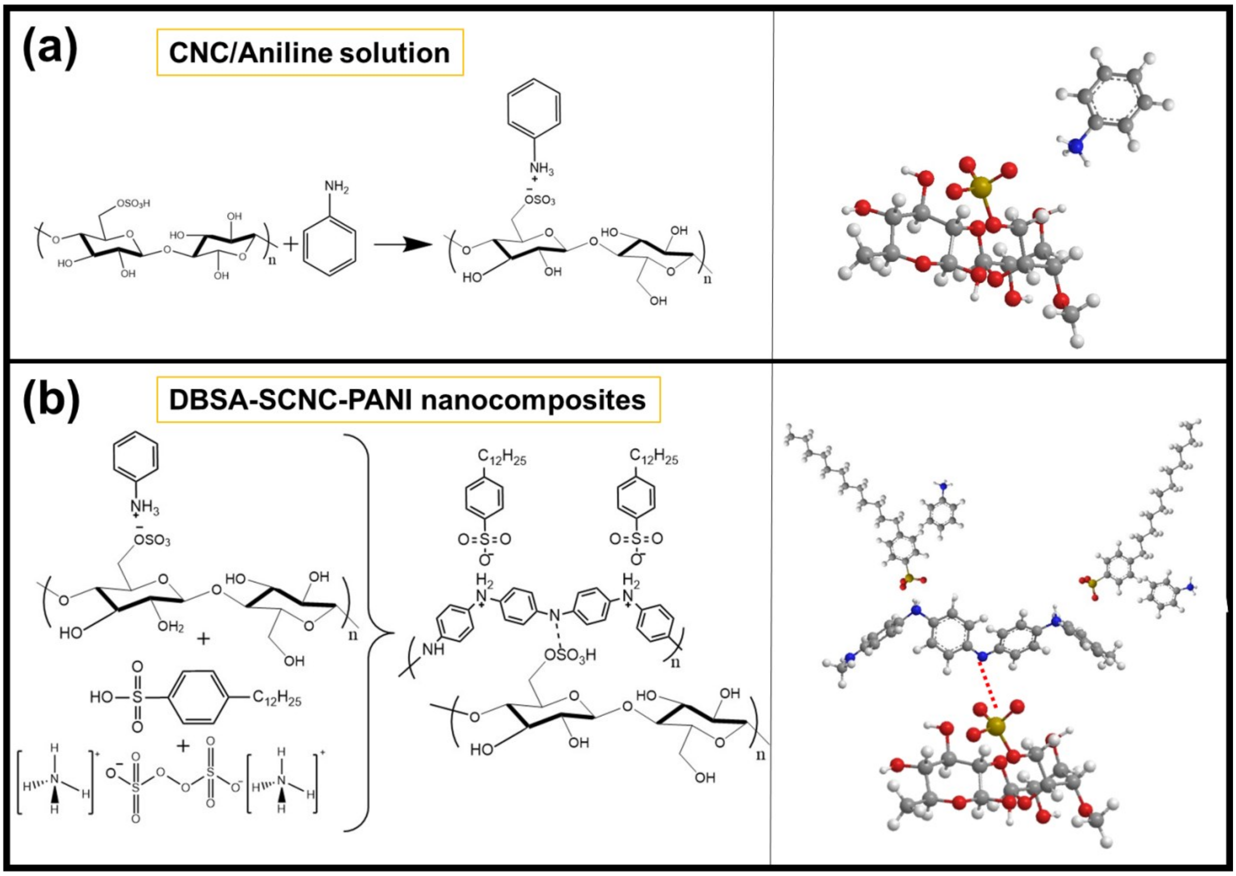

Figure S6. Polymerization of SCNC-ANI by adding DBSA to fabricate the DSP nanocomposites.

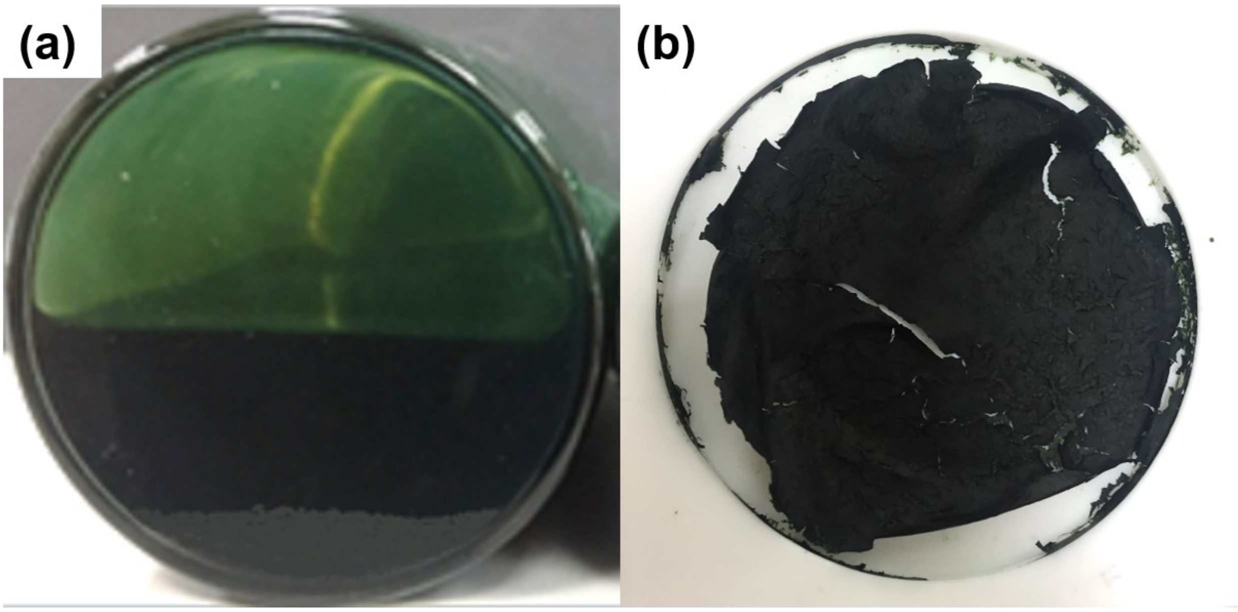

Figure S7. Photographs of CNC/ANI after adding DBSA through emulsion polymerization shows (a) insolubility issues and (b) broken film with large particle aggregation.
